# Expression of human Bcl-xL (Ser49) and (Ser62) mutants in *Caenorhabditis elegans* causes germline defects and aneuploidy

**DOI:** 10.1371/journal.pone.0177413

**Published:** 2017-05-08

**Authors:** Prasamit Saurav Baruah, Myriam Beauchemin, J. Alexander Parker, Richard Bertrand

**Affiliations:** 1 Centre de recherche, Centre hospitalier de l’Université de Montréal (CRCHUM), Montréal (Québec) Canada; 2 Institut du cancer de Montréal, Montréal (Québec) Canada; 3 Département de neurosciences, Université de Montréal, Montréal (Québec) Canada; 4 Département de médecine et Spec. médicales, Université de Montréal, Montréal (Québec) Canada; East Carolina University, UNITED STATES

## Abstract

An interesting feature of Bcl-xL protein is the presence of an unstructured loop domain between α1 and α2 helices, a domain not essential for its anti-apoptotic function and absent in CED-9 protein. Within this domain, Bcl-xL undergoes dynamic phosphorylation and dephosphorylation at Ser49 and Ser62 during G2 and mitosis in human cells. Studies have revealed that when these residues are mutated, cells harbour mitotic defects, including chromosome mis-attachment, lagging, bridging and mis-segregation with, ultimately, chromosome instability and aneuploidy. We undertook genetic experiments in *Caenorhabditis elegans* to understand the importance of Bcl-xL (Ser49) and (Ser62) *in vivo*. Transgenic worms carrying single-site S49A, S62A, S49D, S62D and dual site S49/62A mutants were generated and their effects were analyzed in germlines of young adult worms. Worms expressing Bcl-xL variants showed decreased egg-laying and hatching potency, variations in the length of their mitotic regions but not of their transition zones, appearance of chromosomal abnormalities at their diplotene stages, and increased germline apoptosis, with the exception of the S62D variants. Some of these transgenic strains, particularly the Ser to Ala variants, also showed slight modulations of lifespan compared to their controls. In addition, RNAi experiments silencing expression of the various Bcl-xL variants reversed their effects *in vivo*. Our *in vivo* observations confirmed the importance of Ser49 and Ser62 within Bcl-xL loop domain in maintaining chromosome stability.

## Introduction

Core components of the cell death machinery, identified by genetic and biochemical studies, are well-conserved across eukaryotes, from nematodes to mammals. First identified in *Caenorhabditis elegans* (*C*. *elegans*), *ced-9* is required to protect healthy cells from apoptosis [1]. Initially reported at t(14;18) chromosomal translocation in follicular lymphomas [2], human *BCL2* gene, was latter ascertained to be an ortholog of *ced-9*, whose expression plays a key role in controlling cell death [3]. *BCL2* is the founding member of a large family of genes and proteins, now referred to as the Bcl-2 family [4, 5]. Transient expression of human Bcl-2 in *ced-9* loss-of-function *C*. *elegans*, reduces cell death during nematode development and interacts with the cell death machinery of the worms, revealing the highly-conserved structure and function of these proteins among various species [6, 7]. Indeed, anti-apoptotic proteins, e.g., mammalian Bcl-2, Bcl-xL [8] and *C*. *elegans* CED-9 share structural homology in terms of their Bcl-2 homology (BH) domains that control their apoptosis-regulating functions [9, 10]. However, Bcl-2 and Bcl-xL proteins contain an additional domain, an unstructured loop domain between α1 and α2 helices, a protein domain that is not essential for their anti-apoptotic functions and absent in CED-9 protein [11–15].

Studies have revealed that 2 serine residues within the unstructured loop domain of human Bcl-xL, Ser49 and Ser62, are subjected to cell cycle-dependent, dynamic phosphorylation when cells are subjected to various stresses, but also during normal cell cycle progression [16–23]. Bcl-xL undergoes cell cycle-dependent phosphorylation on Ser49, and accumulates in centrosomes in G2 phase, particularly during DNA single- and double-strand break-mediated G2 arrest [20]. Bcl-xL(Ser49) is rapidly dephosphorylated in early mitotic phases and is re-phosphorylated during telophase/cytokinesis by Polo kinase 3 (Plk3) [20]. Phospho-Bcl-xL(S49) is found in conjunction with microtubule-associated dynein motor proteins and in mid-zone bodies during telephase/cytokinesis [20].

Bcl-xL is also phosphorylated at Ser62 by Plk1 and mitogen-activated protein kinase 9/c-jun N-terminal kinase 2 (Mapk9/Jnk2) during normal cell cycle progression at G2 and after DNA damage [21]. At G2, phospho-Bcl-xL(Ser62) accumulates in nucleolar structures including nucleoli and Cajal bodies and co-localizes with cyclin-dependent kinase 1 (Cdk1) [21]. During mitosis, Bcl-xL(Ser62) is strongly phosphorylated by Plk1 and Mapk14/stress-activated protein kinase p38α in prophase, prometaphase, metaphase and the anaphase boundary, while it is dephosphorylated in telophase and cytokinesis [22]. At mitosis, phospho-Bcl-xL(Ser62) localizes in centrosomes with γ-tubulin, and in the mitotic cytosol with some spindle-assembly checkpoint (SAC) signalling proteins, including Plk1 and the Mad2-, BubR1-, Bub3- and Cdc20-bound complexes [22].

In normal human fibroblasts or human tumor cells, expression of the phosphorylation mutants Bcl-xL(S49A) or (S49D), Bcl-xL(S62A) or (S62D), or dual Bcl-xL(S49/62A) or (S49/62D) has no significant effect on the apoptosis rate, but leads to mitotic defects associated with chromosome mis-attachement, lagging, bridging, mis-segregation, cytokinesis failure and aneuploidy [22, 23]. These findings conferred novel functions to the protein linked with the unstructured loop domain of Bcl-xL. In the present study, to better characterize this function *in vivo*, we hypothesize that, although CED-9 lacks Bcl-xL's loop domain, introduction of Bcl-xL(Ser49) and (Ser62) mutants in *C*. *elegans* has dominant effects and can cause proliferating germline cell defects as well as aneuploidy.

## Materials and methods

### Worm-handling

Worms were manipulated according to standard methods and maintained at 15°C in nematode growth media (NGM) plates seeded with OP50 *Escherichai coli* (*E*. *coli*), unless otherwise indicated for specific assays.

### *C*. *elegans* genetic manipulations and molecular assays

Influenza hemagglutinin (HA)-tagged human Bcl-xL (wt) and single-site (S49A, S49D, S62A, S62D) or dual-site (S49/62A, S49/62D) mutant cDNAs containing the *ced-9* promoter and 3'UTR sequences were generated with the MultiSite Gateway^TM^ recombinational cloning system (Thermo Fisher Scientific, Waltham MA), and inserted into the final destination plasmid pCFJ150-pDESTttTi5605[R4-R3] generated in Dr Erik Jorgensen laboratory (University of Utah, Salt Lake City, UT) and obtained from Addgene (Cambridge, MA). The strain EG4322 (ttTi5605 II; *unc-119(ced9)* III) was studied, and transgenic worms were generated by Knudra Trangenics (Murray, UT) with the mos1-mediated single copy insertion (MosSCI) method [24]. Transgenic worm insertions were tested by PCR with primers, 5’-ATGGGCCGCATCTTTTAC-3’ and 5’-TCATTTCCGACTGAAGAG-3’. All insertions were validated by DNA sequencing. Quantitative reverse transcriptase PCR (qRT-PCR) was performed on the transgenic worms and on 250–300 extracted gonads, with *pmp-3* as a control of constitutive gene expression[25]. The oligonucleotide primers for *pmp-3* were 5’-GTTCCCGTGTTCATCACTCAT-3’ and 5’-ACACCGTCGAGATGTAGA-3’. The oligonucleotide primers for *BCL-XL* (wt) and mutants were 5’-GGTAAACTGGGGTCGCATTG-3’ and 5’-GTTCTCCTGGATCCAAGGCT-3’, and for *ced-9* were 5'-ACGGTTGGAAATGCACAGAC-3' and 5'-TGTTCCCAGTTGTTGCG-3'. To monitor apoptotic body formation, transgenic worms expressing CED-1:GFP in sheath cells (strain HR1459 (bcls39[lim7:ced-1:GFP;lim-15(+)]V), generously provided by Dr. Jean-Claude Labbé (Institut de recherche en immunologie et cancer, Montréal QC), were crossed with transgenic male worms expressing Bcl-xL (wt) and mutants. Homozygous progeny were screened and processed for assays. For the RNA interference (RNAi) assays, *BCL-XL* open reading frame (ORF) cDNA was cloned into the vector L4440-gateway, generated in Guy Caldwell laboratory (University of Alabama, Tuscaloosa, AL and obtained from Addgene, and transformed into the HT115 competent bacteria, generously donated by Dr. Jean Claude Labbé (Institut de recherche en immunologie et cancer, Montréal QC). The transformed bacteria colonies were selected and PCR sequenced. The worms were grown in plates containing IPTG (1 mM) and HT115 containing L4440-*BCL-XL* vector. The transgenic worms were grown up to 5 generations prior to perform experiments to achieve high penetrance of *BCL-XL* silencing. Empty L4440 vector was used as negative control in the RNAi assays. All experiments were performed in parallel with wild type control N2 worms.

### Progeny count

Adult worms were collected and washed with M9 buffer to remove bacterial contamination. Worm pellets were treated with freshly-prepared 0.5 ml NaOH(5N) mixed with 1 ml commercial bleach for 10 min. Samples were briefly vortexed at 2-min interval, then centrifuged for 30 s at 1,300 g to pellet the released eggs. The pelleted eggs were washed with sterile H_2_O, volume reduced to 100 μl and then plated in fresh NGM. When larvae reached the L4 stage, they were placed individually in fresh NGM plates. Progeny count was started 12 h latter, once they reached the young adult stage. Eggs layed were counted for the first 3 days of adulthood, and hatched eggs were counted every 8 h. Worms were transferred into fresh NGM plates every day.

### Gonad staining, MR and TZ measurements, and germ cell count

Young adult worms (12 h from the L4 larvae stage) were washed with M9 buffer and placed on glass slides. Worms were fixed with 100% cold methanol (-20°C) for 30 s and washed with M9 buffer. Gonads were stained with DAPI at a concentration of 1.0 ng/ml for 3 min and washed with phosphate buffered saline (PBS) prior to microscopy. MR/TZ or TZ/pachytene boundaries were well marked under the microscope. Germ cell numbers in the gonad were also determined by first marking MR/TZ or TZ/pachytene with the microscope, and then counting nuclei through germline width. Images were generated with a Zeiss Axio Observer Z1 automated microscope equipped with Axiovision software (v4.8.2).

### Fluorescence staining and analysis

Young adult worms were dissected to expose their gonads and fixed on polylysine-coated slides with 100% cold methanol (4 min), followed by 100% cold acetone (4 min). For immunofluorescence staining, the samples were first treated with a blocking solution containing 10% donkey serum (Jackson Immuno Research Laboratories, Inc., West Grove PA) for 1 h at room temperature, then stained overnight at 4°C in an humidified chamber with RAD-51 (14B4) Alexa Fluor (R) 488 Ab (Novus Biologicals, Oakville ON), in PBS solution containing 0.5% (v/v) Triton X-100, 1 mM EDTA pH 8.0, 0.1% (w/v) bovine serum albumine (BSA) and 0.05% (w/v) sodium azide. Samples were also stained with DAPI at a concentration of 1.0 ng/ml for 3 min. Images were generated with a Nikon microsystem mounted on a Nikon Eclipse E600 microscope with a photometric Cool-Snap HQ2 camera and Nikon NIS-Elements software 9 (v 3.8AR) or with a Zeiss Axio Observer Z1 automated microscope and Axiovision software (v4.8.2). Images were analysed by Image J software (v1.49), a Java-based processing program developed by the National Institutes of Health (USA).

### Western blots

Worms were collected in maintenance (M9) buffer and washed several times until no visible bacterial contamination was observed in the washing M9 buffer. Worm pellets were froozen overnight at -80°C, and pellets were lysed with buffer containing 150 mM NaCl, 50 mM Tris:HCl, pH 7.4, 1% (v/v) Triton X-100, 0.1% (w/v) sodium dodecyl sulfate (SDS), 1% (w/v) sodium deoxycholate (w/v), 10 μg/ml leupeptin, 10 μg/ml chymostatin and 10 μg/ml pepstatin A. Pellets were directed through 271/2G syringes (10-fold), sonicated and centrifuged at 16,000g. Protein in supernatants were collected and their concentrations measured by bicinchoninic acid assays as described in the manufacturer's protocol (Thermo Fisher Scientific). Bcl-xL(54H6) rabbit monoclonal Ab (Cell Signaling Technology, Whitby, ON), and actin (AC-15) mouse monoclonal Ab (Abcam Inc., Cambridge, MA) were tested in this study. Peroxidase-labeled secondary Ab were detected by enhanced chemiluminescence with reagent set from GE Healthcare Life Science (Mississauga, ON) or SuperSignal WestPico chemiluminescence substrates (Thermo Scientific, Rockford, IL). Densitometry analysis, were analysed by Image J software (v1.49), a Java-based processing program developed by the National Institutes of Health (USA).

### Lifespan assay

Lifespan assays were peformed at 20°C. Briefly, 45 to 50 L4 hermaphrodite transgenic larvae, and N2 (wt) larvae were placed in 400 μM 5'flurodeoxyuridine-NGM plates in triplicate, for 6 days. Day 1 was defined as the day when the worms reached adulthood. Worms were scored every 1 to 3 days. On the 6th day, they were transferred to fresh NGM plates. Strains were considered to have lost viability if they exhibited arrested development or died.

## Results

### Expression of human Bcl-xL variants in *C*. *elegans*

**Fig 1:**
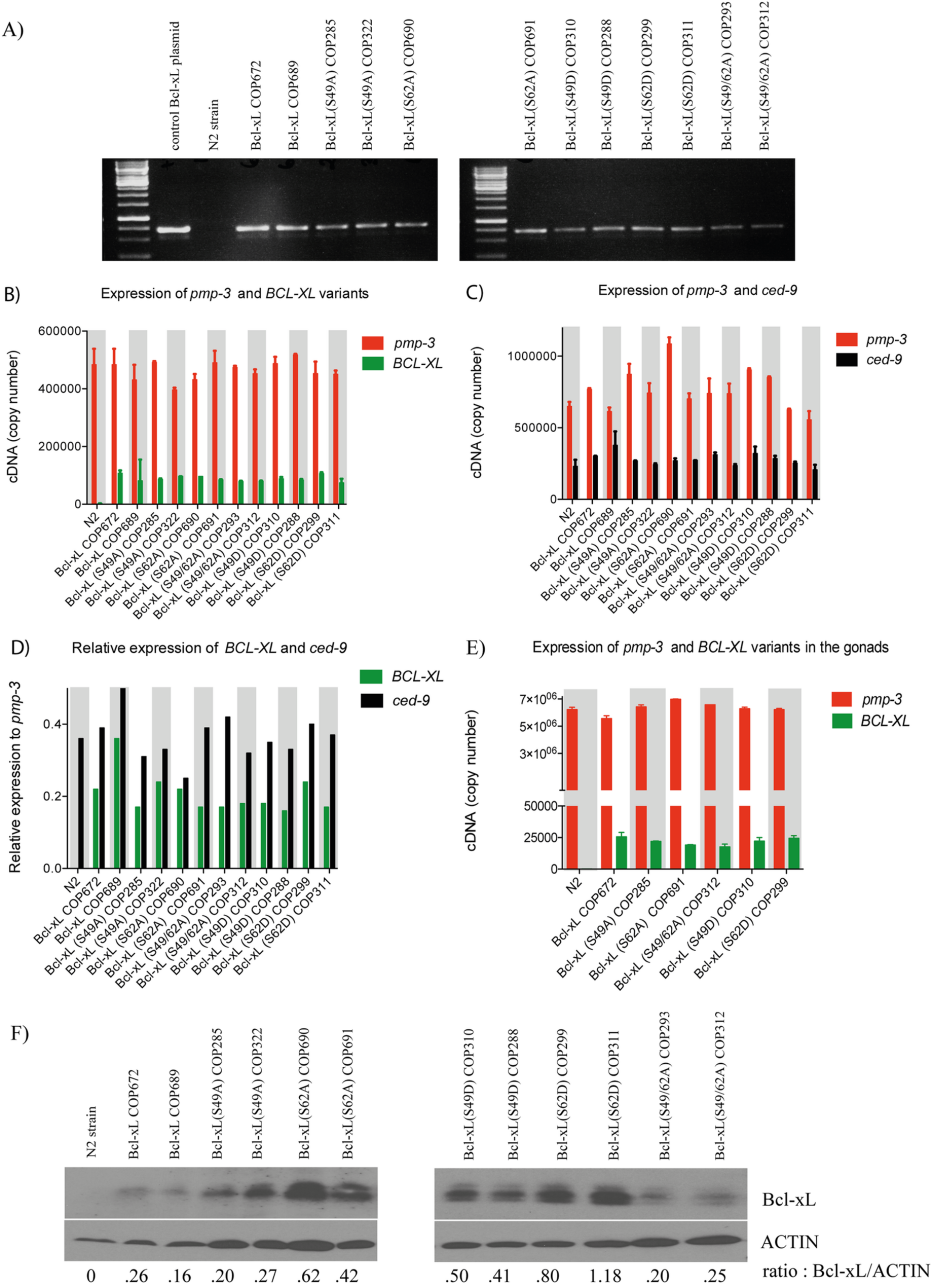
Expression of Bcl-xL (wt) and Bcl-xL variants in transgenic worms. **A**) Ethidium bromide-stained PCR products obtained by genomic DNA amplification reactions. mRNA expression levels assessed by qRT-PCR of **B)** *BCL-XL* and *pmp-3*, **C)** *ced-9* and *pmp-3* and **D)** relative expression of *BCL-XL* and *ced-*9 in the transgenic worms. In **E)** mRNAs were extracted from 250–300 gonads of selected strains and expression levels assessed by qRT-PCR of *BCL-XL* and *pmp-3*. Results from 2 independent determinations. Bars are means ± variations. **F)** Immunoblots of Bcl-xL variant expression in various transgenic strains and control worms. Actin expression is shown as control. Bcl-xL/ACTIN ratio are indicated.

Several strains of transgenic worms containing human Bcl-xL wild-type (wt) and single-site (S49A, S49D, S62A, S62D) or dual-site (S49/62A) mutants were generated (S1 Table). First, 2 strains for each Bcl-xL variant and Bcl-xL wt (COP672 and COP689) were used, and N2 (wt) worms served as controls. Transgenes were confirmed by polymerase chain reaction (PCR) assays of genomic DNA (Fig 1A). mRNA levels were evaluated by quantitative reverse-transcriptase (q-RT)-PCR using *pmp-3* expression as referencence gene [25], and all transgenic worms expressed similar levels of *BCL-XL* variant mRNAs (Fig 1B). *Ced-9* mRNA levels were monitored in parallel in these experiments (Fig 1C). Overall, *BCL-XL* mRNA expression was found slightly lower compared to *ced-9* mRNA expression, with *BCL-XL* / *ced-9* ratios ranging from 0.56 to 0.88 (Fig 1D). On randomly selected strains representing each variant, mRNA levels in the gonads were also evaluated by quantitative reverse-transcriptase (q-RT)-PCR using *pmp-3* expression as referencence gene (Fig 1E). Expression at the protein level was confirmed by Western blotting (Fig 1F). Overall, Bcl-xL and Bcl-xL variant protein expression was found variable, with some strains expressing higher protein level compared to Bcl-xL (wt), perhaps suggesting differential stability among these proteins.

### Mutations within the loop domain of Bcl-xL affect *C*. *elegans* progeny fecundity

N2 (wt) hermaphrodites under laboratory conditions released embryos from the uterus during their development, and these eggs were easily counted. To determine whether the expression of Bcl-xL (wt) and mutants in *C*. *elegans* affects their progeny, eggs were counted and compared among various transgenic strains. Synchronized L4 hermaphrodite transgenic larvae were plated individually, and eggs laid were counted over a 3-day period once they have reached adulthood (about 12 h after plating).

**Fig 2:**
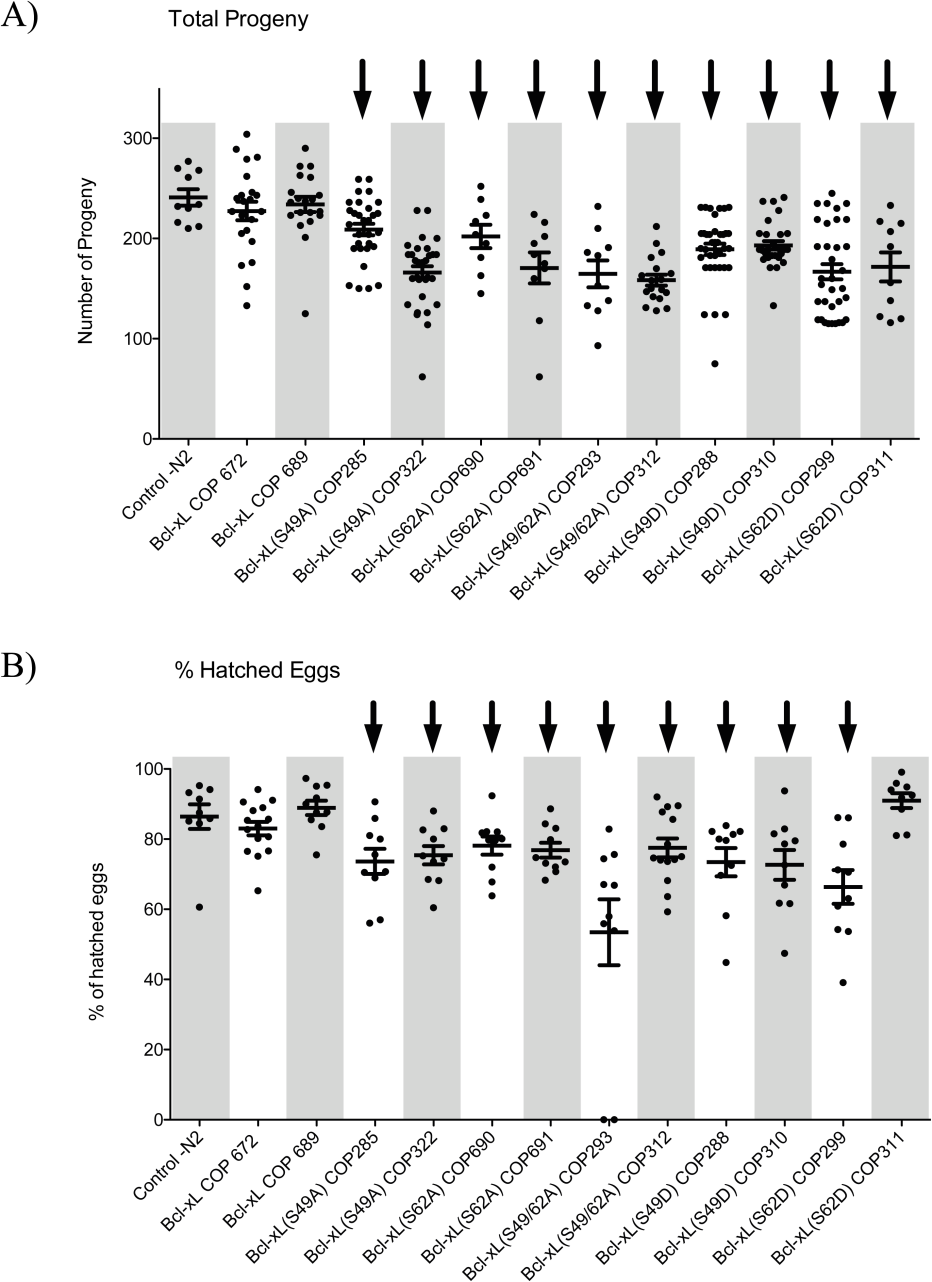
Effects of Bcl-xL (wt) and Bcl-xL variants on *C*. *elegans* progeny fecundity. Number of **A)** total viable progeny, and **B)** percentages of eggs hatched in various transgenic strains and control worms. Each point show in the graphs represents data obtained from a single worm. Bars are means ± s.d. Arrows on top indicate statistical significance with *p<0*.*05* when compared to N2 control.

The transgenic strains expressing Bcl-xL (wt) showed no significant difference in egg-laying potency (Fig 2A) and in percentage (%) of eggs that hatched (Fig 2B) compared to N2 (wt) worms. The number of eggs laid by transgenic worms expressing Bcl-xL mutants was decreased significantly compared to Bcl-xL (wt)-expressing worms and N2 (wt) worms (Fig 2A). When these eggs were followed, the % that hatched was significantly affected across all the mutants with the exception of Bcl-xL (S62D) variant strain 311, that may reflect strain and/or animal variations. Overall, embryonic lethality is presented in S1 Fig. Progeny difficulties might result from a vulva defect rather than fertility defects.

### Mutations within the loop domain of Bcl-xL causes nuclear morphology aberrations in the gonads

Preliminary and simple chromatin staining of germlines revealed increased numbers of aberrant cells harboring condensed chromatin and/or fragmented chromatin and/or doublet cells as well as global spatial disorganization of germline alignment in the transgenic strains expressing the Bcl-xL Ser to Ala mutants (S2 Fig). These preliminary observations were then analyzed in more detail in selected strains in which mRNA expression in the gonads was confirmed, and in which, Bcl-xL/ACTIN protein ratio range from .26 to .80.

**Fig 3:**
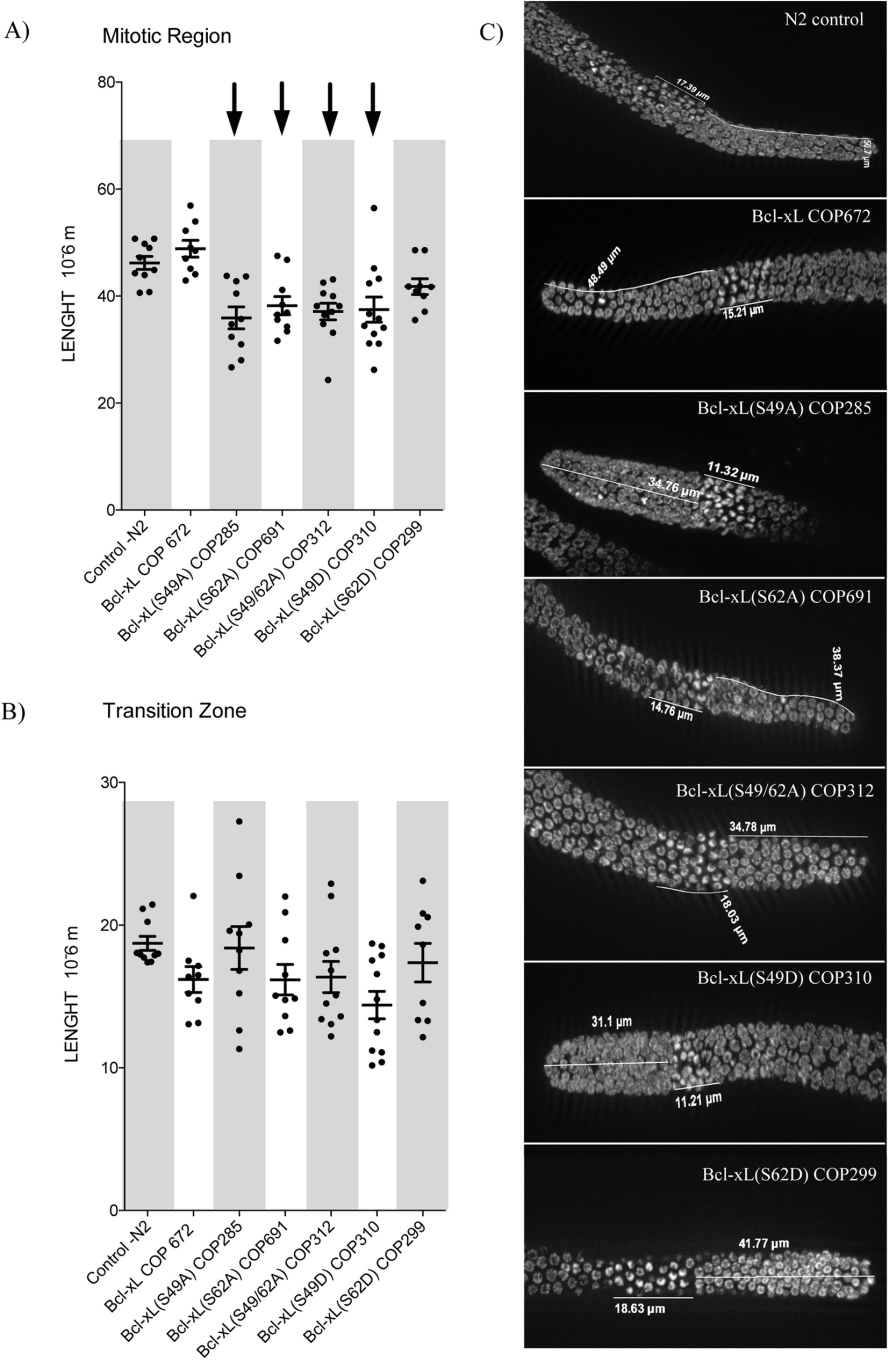
Effects of Bcl-xL (wt) and Bcl-xL variants on mitotic region and transition zone length in *C*. *elegans* gonads. Length (μm) of **A)** mitotic regions and **B)** transition zones of the gonads. Each point in the graphs represents data obtained from a single worm; both MR and TZ were determined from the same worms. Bars are means ± s.d. Arrows on top indicate statistical significance with *p<0*.*05* when compared to N2 control **C)** low-magnification images of DAPI-stained cells.

To determine if Bcl-xL mutations at Ser49 and Ser62 affect the process of mitosis, we analyzed the germline in the transgenic worms. The *C*. *elegans* germline contains an anatomically-restricted mitotic cell population that persists throughout life and is thought to be self-renewing [26]. The mitotic region (MR) in the gonads showed reduced lengths in all Bcl-xL variants compared to Bcl-xL (wt)-expressing worms or N2 control worms (Fig 3A) with the exception of Bcl-xL (S62D) variant. In contrast, the transition zones (TZ) in the gonads presented no overall difference in all Bcl-xL variants compared to Bcl-xL (wt)-expressing worms or N2 control worms (Fig 3B). Representative micrographs are reported in Fig 3C.

### Mutations within the loop domain of Bcl-xL cause germ line aneuploidy

**Fig 4:**
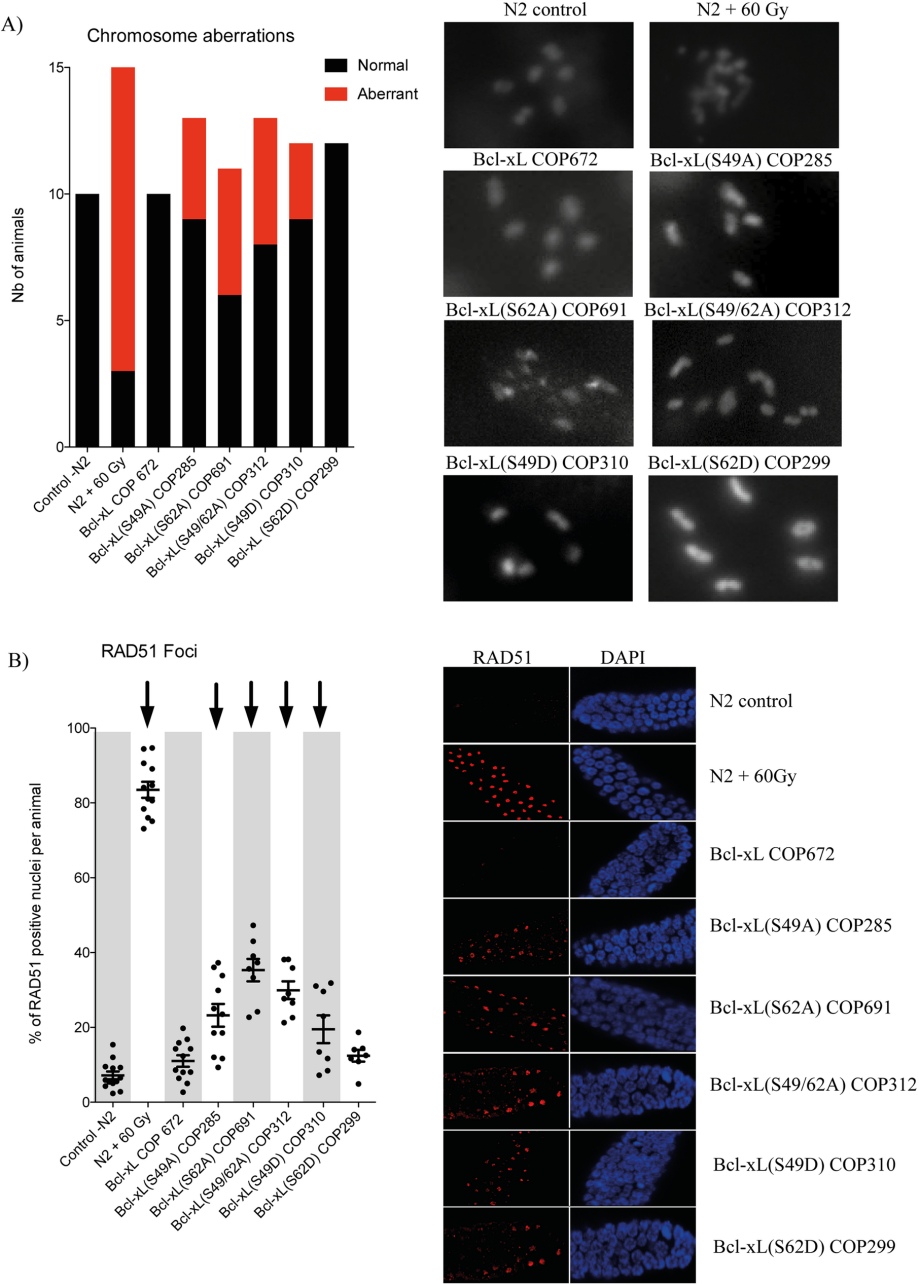
Effects of Bcl-xL (wt) and Bcl-xL variants on *C*. *elegans* chromosome stability and aneuploidy. **A)** The graph on left shows the number of cells with normal (black) and abnormal genotype (red). Right panels: images of DAPI-stained structures observed at the diplotene stages at proximal gonads. **B)** % of cells showing RAD51-associated nuclear foci in germline nuclei (left graph). Each point in the graph represents data obtained from a single worm. Bars are means ± s.d. Arrows on top indicate statistical significance with *p<0*.*05*. Right images: typical low-magnification images of immunofluorescence illustrating RAD51-associated nuclear foci (red) and DAPI-stained cells (blue). N2 animals subjected to high-dose radiation (60 Gy) were collected 4 hrs post-irradiation and used as strong reference controls in **A)** and **B).**

Chromosome mis-alignment, lagging or bridging, mis-segregation and cytokinesis failure are major defects that could occur during mitosis and confer chromosome instability as well as aneuploidy [27, 28]. Cells will respond in various ways including cell cycle checkpoint activation, cell cycle arrest, premature senescence or cell death and, in mammal cells, they could also enter into an immortalization or tumorigenesis path, depending on particular cellular and environmental contexts [29–31]. To evaluate the effect of the various Bcl-xL variants on chromosome stability in *C*. *elegans*, chromosomes were analyzed at the diplotene stage in control and transgenic worms. In N2 control and in Bcl-xL (wt) worms, 6 pairs of chromosomes were clearly visible in the diplotene stage at proximal gonad. However, various Bcl-xL mutants, with the exception of Bcl-xL (S62D) variant, underwent aneuploidy and/or chromosome fragmentation compared to N2 control and Bcl-xL (wt) strains (Fig 4A). RAD51-associated nuclear foci in the germline confirmed the presence of DNA double-strand breaks in transgenic strains expressing Bcl-xL mutants, with the exception of the Bcl-xL (S62D) variant (Fig 4B). N2 animals subjected to high-dose radiation (60 Gy) were collected 4 hrs post-irradiation and used as strong reference controls (Fig 4A and 4B).

### Mutations within the loop domain of Bcl-xL cause increased apoptosis in the gonads

**Fig 5:**
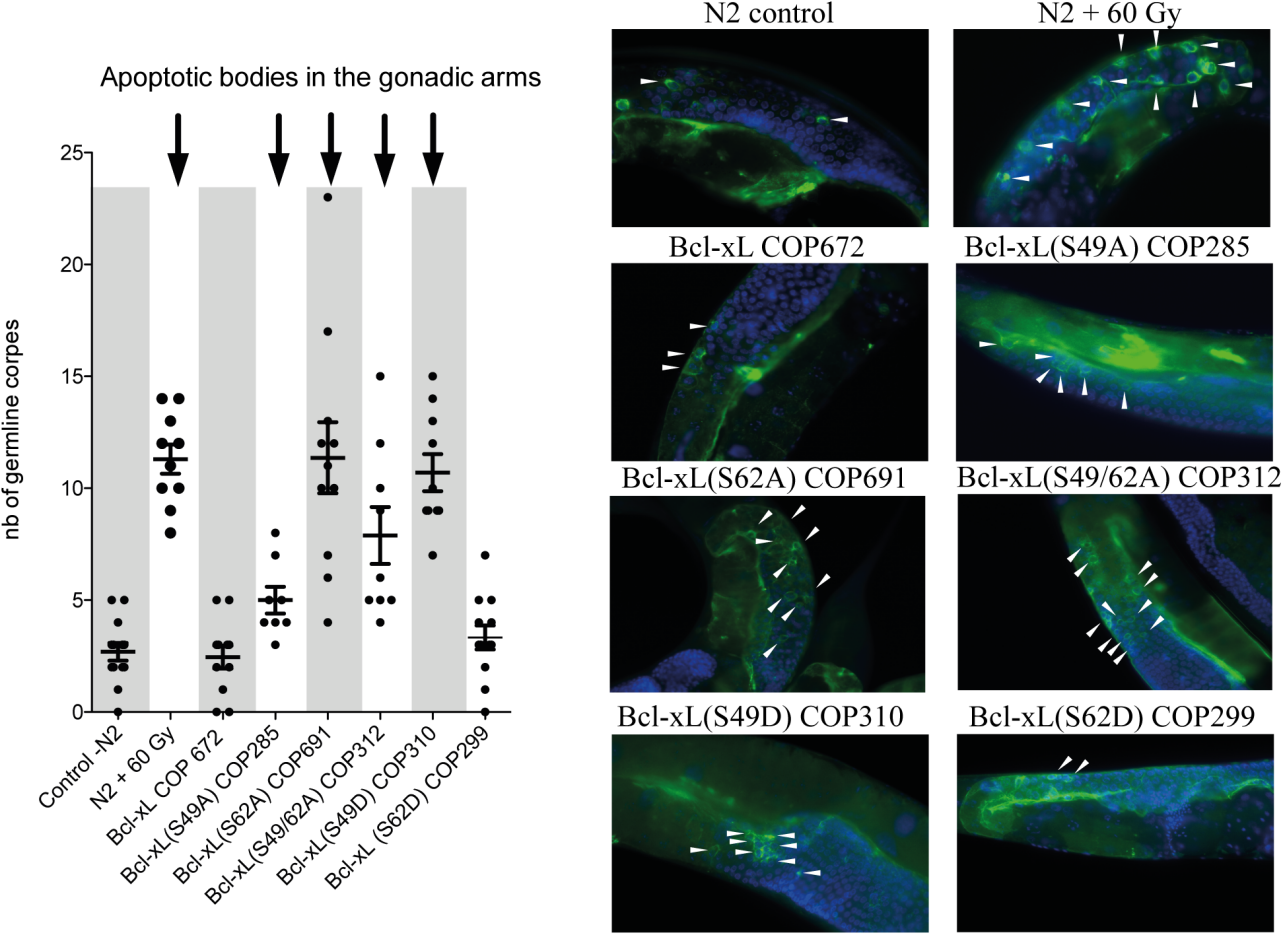
Effects of Bcl-xL (wt) and Bcl-xL variants on germline apoptosis. Left graph: Number of cells showing apoptotic corpses. Left panel, each point in the graphs represents data obtained from a single worm. Bars are means ± s.d. Arrows on top indicate statistical significance with *p<0*.*05*. Right panels: Typical low-magnification images of CED1:GFP expression in various transgenic strains and control worms. N2 animals subjected to high-dose radiation (60 Gy) were collected 4 hrs post-irradiation and used as reference controls.

Finally, to assess apoptosis in germlines, we took advantage of a CED-1:GFP strain [32] by crossing it with our transgenic worms. CED-1, expressed in sheath cells, is a phagocytic receptor that initiates pathways for degrading engulfed apoptotic cells and is thus a good indicator apoptotic bodies [33]. With the exception of the Bcl-xL (S62D) variant, worms expressing Bcl-xL mutants showed significantly increased apoptotic bodies compared to those expressing Bcl-xL (wt) and the N2 (wt) worms (Fig 5). Again, N2 animals subjected to high-dose radiation (60 Gy) were collected 4 hrs post-irradiation and used as strong reference controls (Fig 5).

### Longevity changes due to the expression of Bcl-xL mutants

**Fig 6:**
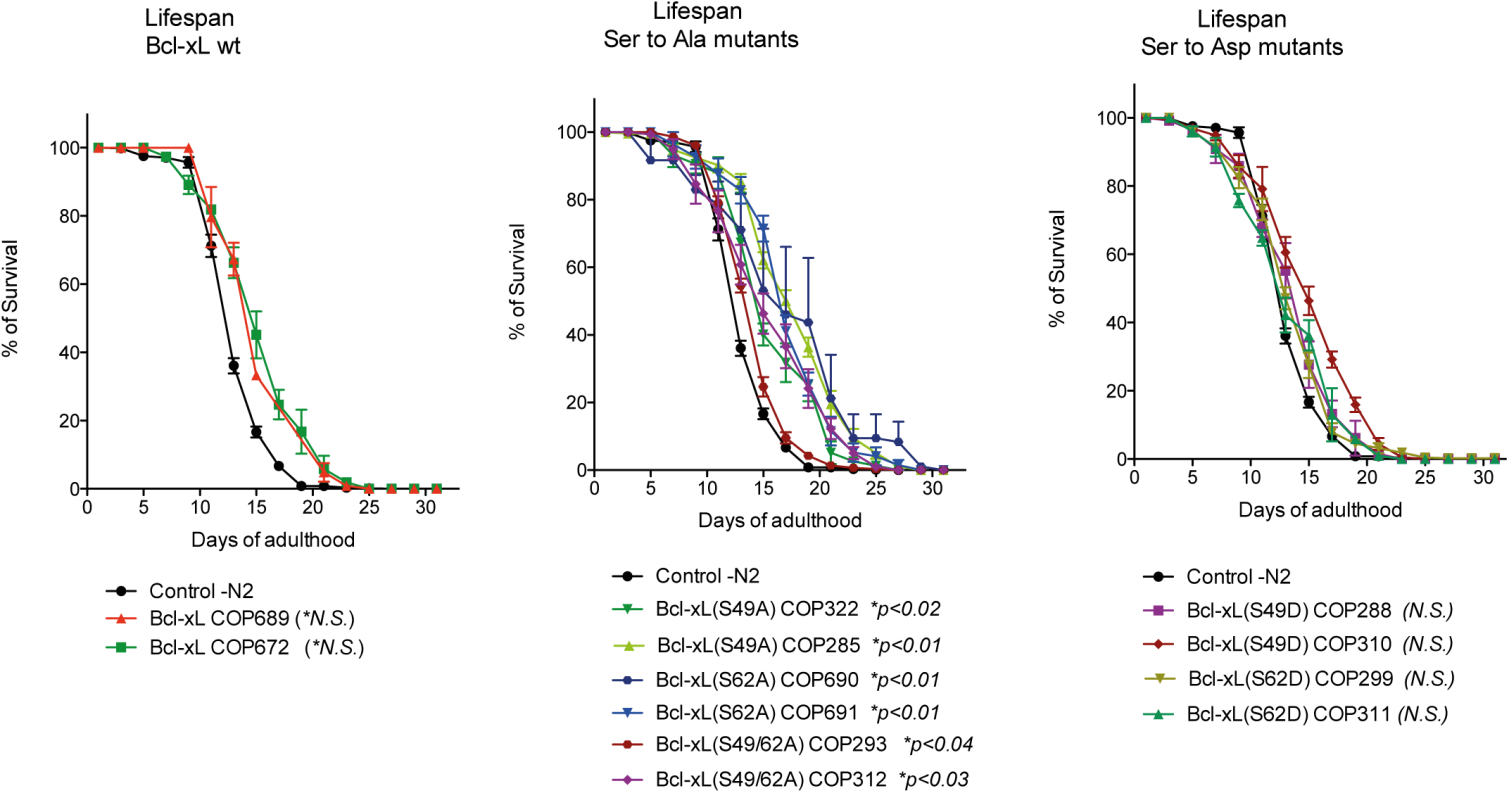
Effects of Bcl-xL (wt) and Bcl-xL variants on *C*. *elegans* lifespan. Lifespan kinetics of Bcl-xL(wt)-expressing worms (left graph), Bcl-xL (Ser to Ala) variants (middle graph) and Bcl-xL (Ser to Asp) variants (right graph). Data obtained from 2 independent triplicate experiments (n = 6). Bars are means ± s.d. Statistical significance is indicated below the graphs.

The *C*. *elegans* N2 strain has an average lifespan of around 2–3 weeks at 20°C [34]. N2 and Bcl-xL (wt) worms showed no significant differences in their lifespan (Fig 6). Strains expressing Ser to Asp variants also presented no significant differences compared to N2 controls. In contrast, overall lifespan was significantly increased in strains expressing Ser to Ala variants (Fig 6).

### Reversion of the phenotypes

**Fig 7:**
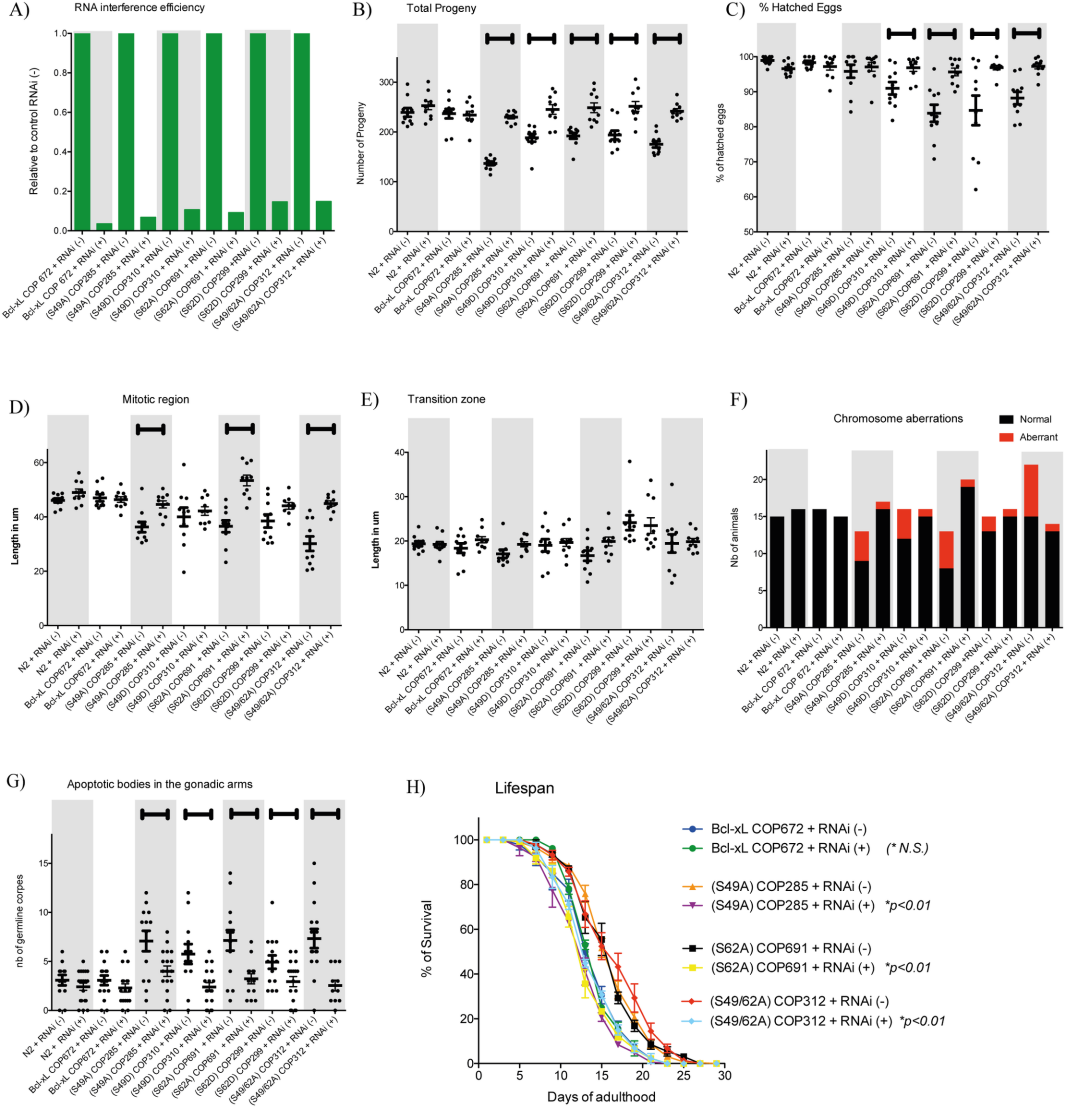
Effects of silencing the expression of *BCL-XL* variants in various transgenic strains and control worms. **A)** RNAi efficiency, **B)** total viable progeny and **C)** percentages of eggs hatched, **D)** mitotic regions and **E)** transition zones, **F)** chromosome stability and aneuploidy, **G)** germline apoptosis of the gonads and **H)** lifespan. Each point in graph (**B to G**) represent data obtained from single worm. Bars are means ± s.d. Brackets on top of graph **(B to G)** indicate statistical significance with *p<0*.*05* between worms subjected to control RNAi (-) and *BCL-XL* RNAi (+). Statistical significance for **H)** is indicated in graph legend. Typical micrographs are showed in S3 and S4 Figs.

Finally, to confirm that the phenotypes observed were due to the expression of Bcl-xL variants in transgenic *C*. *elegans*, a serie of RNA interference experiments were conducted. Silencing *BCL-XL* mRNA variant expression (Fig 7A) in the transgenic worms reversed the phenotypes, including effects on germline fecundity measured as egg-laying and egg-hatching potency (Fig 7B and 7C), mitotic region length (Fig 7D) and transition zone length (Fig 7E), germline aneuploidy (Fig 7F), apoptotic corpse appearance in the gonads (Fig 7G) and lifespan for the Ser to Ala variants (Fig 7H). Typical micrographs are showed in S3 and S4 Figs.

A summary of observations is presented on Table 1.

**Table 1:** Summary of observations.

STRAIN	COP672 WT	COP285 S49A	COP691 S62A	COP312 S49/62A	COP310 S49D	COP299 S62D
mRNA in gonads	Yes	Yes	Yes	Yes	Yes	Yes
Protein expression relative to Bcl-xl (wt)	1.00	0.77	1.61	0.96	1.92	3.07
Total progeny	normal	reduced	reduced	reduced	reduced	reduced
% hatched eggs	normal	reduced	reduced	reduced	reduced	reduced
Mitotic region	normal	reduced	reduced	reduced	reduced	reduced
Transition zone	not different	not different	not different	not different	not different	not different
Chromosome aberration	No	Yes	Yes	Yes	Yes	No
RAD51 Foci	No	Yes	Yes	Yes	Yes	No
Apoptotic bodies	normal	increased	increased	increased	increased	normal
Lifespan	normal	increased	increased	increased	normal	normal

## Discussion

The loop domain between α1 and α2 helices of Bcl-xL in higher organisms may be due to gain-of-function domain through evolution. In most studies, the loop domain is not necessary for the Bcl-xL's anti-apoptotic activity in mammallian cells [11–15, 20–22]. However, mutations within Bcl-xL (Ser49) and (Ser62) residues lead to chromosome instability and aneuploidy in human cells [22, 23]. *C*. *elegans* CED-9, the only anti-apoptotic ortholog of the Bcl-2 family, does not contain the Bcl-xL loop domain. A search for sequence homology in the *National Center for Biotechnology Information* (NCBI) and *WormBase* failed to identify other putative proteins possibly containing a similar domain in *C*. *elegans*. The presence, or absence, of a similar conserved function within *C*. *elegans* protein remains to be discovered. [35]. In this study, Bcl-xL phosphorylation in *C*. *elegans* was not determined; however the presence of conserved PLK-1 and PLK-3 activities in *C*. *elegans* raises a probability of similar phosphorylation at Ser62 and Ser49 alike human cells. Indeed, PLK-1 and PLK-3 are well conserved in *C*. *elegans* and involved in multiple process of mitosis, including spindle formation, kinetochore-microtubule attachments, sister chromatid separation and cytokinesis [36, 37]. PLK-1 is shown to be involved in nuclear envelope breakdown in the oocyte during meiosis and in mixing maternal and paternal genomes after fertilization. Partial inactivation of PLK-1 caused failure of alignment of chromosomes at metaphase during mitosis and the nuclear membrane remains intact [38]. In contrast, PLK-3 mutations caused delay in chromosome condensation at diakinesis indicating that PLK-3 does not play a major role in meiosis [39].

Although the CED-9 protein lacks the Bcl-xL functional loop domain, we tested whether or not human Bcl-xL (Ser49) and (Ser62) variant expression in *C*. *elegans* exhibits dominant effects on mitotic behaviors, as observed previously in human cells. The proliferative properties of germlines of adult young worms [40], as well as the short and reproducible lifespan of *C*. *elegans* is well-characterized [41]. Expression of mammalian proteins is prevalent in *C*. *elegans* and *viceversa* is prevalent. Expression of human Bcl-2 itself partially prevents apoptosis in *C*. *elegans* [7, 42], whereas CED-9 expression in monkey fibroblast COS cells and embryonic drosophila Schneider's L2 cells reveals co-localization of the 2 proteins, suggesting similar functions [43].

In the present study, we observed that human Bcl-xL (Ser49) and (Ser62) variant expression in *C*. *elegans* interfered with germline fertility, effects that correlated with MR length variations, the appearance of chromosomal aberrations, RAD51-associated foci and increase apoptosis with the exception of Bcl-xL (S62D) variants. It is interesting to note that the Bcl-xL (S62D) strain that express higher protein level compared to other variants, showed undetectable cell division errors (summary in Table 1). Most likely, reduced fecundity could resulted from cell division errors in germlines and embryos, resulting in chromosome aberrations, aneuploidy and augmented apoptosis. MR length variations were seen as being decreased in individual worms and strains bearing Bcl-xL variants. Perhaps defects in mitosis and elevated apoptosis could account for shortened length. All experiments were performed on proliferative germline in the gonads. In the near future, time-lapse imaging on embryos could further document mitotic behaviours and chromosome stability/instability in dividing embryos.

Lifespan modulation also has been observed in Ser to Ala variants, a possible consequence of aneuploidy, DNA damage and increased apoptosis of germlines. Indeed, repeated ultra-violet electromagnetic radiation exposure has been shown to severely reduce lifespan in *C*. *elegans* [44], whereas mutations in the nucleotide excision repair proteins ERCC-1 and XPF-1 extend lifespan in *daf-2* worms. Fecundity also decreased in worms expressing mutant ERCC-1, XPF-1 and XPG-1 compared to wt proteins [45]. In the long-term, it would be interesting to monitor gene expression profiles in various strains to identify genes whose expression could be altered as well.

The exact mechanisms by which Bcl-xL exerts its function on chromosome stability are unknown, but Ser49 and Ser62 are 2 essential residues associated with this activity in human cells. Previous studies have revealed the presence of phospho-Bcl-xL(Ser49) and (Ser62) in centrosomes with γ-tubulin during G2 and mitosis, respectively [20, 22]. In addition, phospho-Bcl-xL(Ser62) interacts in mitotic cytosol with some SAC signaling proteins during prometaphase/metaphase, including the Mad2-, BubR1-, Bub3- and Cdc20-bound complexes [22], while phospho-Bcl-xL(Ser49) is found in mid-zone bodies during telephase/cytokinesis [20]. In *C*. *elegans*, most of these key players in the SAC signaling pathway are functionally and structurally conserved, including MAD1, MAD2, MAD3/BubR1, BUB1, BUB3 and FZY-1/Cdc20 [46]. Proliferating germ cells have functional SAC [47], but SAC function in *C*. *elegans* embryos is unclear due to their lack of apparent mitotic arrest phenotype [48]. SPDL-1, a *C*. *elegans* homolog of kinetochore-specific dynein recruiter protein [49] that senses the microtubule attachment status of kinetochore and functions upstream of MAD1MDF-1, is part of the kinetochore receptor of the MAD1MDF-1–MAD2MDF-2 complex that regulate APC/C activity [49, 50]. Whether or not, and how, human Bcl-xL interplays with these *C*. *elegans* components remains to be elucidated.

Bcl-xL expression in human cancers is often associated with poor prognosis and chemotherapy resistance [51–53]. Current efforts are being made to develop and test new drugs targeting the conventional BH1-, BH2-, BH3-forming hydrophobic pocket domain of Bcl-2 anti-apoptotic members including Bcl-xL [54–59]. Future perspectives should also focus on the loop domain of Bcl-xL and Bcl-2 for therapeutic evaluation. These *in vivo* transgenic strains will be an important tool to screen and evaluate the effects of future putative new compounds targeting this function.

## Supporting information

S1 Fig Effects of Bcl-xL (wt) and Bcl-xL variants on *C*. *elegans* embryonic lethality. Percentages of embryonic lethality in various transgenic strains and control worms. Each point show in the graphs represents data obtained from a single worm. Bars are means ± s.d. Arrows on top indicate statistical significance with *p<0*.*05* when compared to N2 control.
(TIF)

S2 Fig Effects of Bcl-xL (wt) and Bcl-xL variants on the gonads. Graph showing the percentage of aberrant cells per worms and images of DAPI-stained germlines of various trangenic strains and control worms. Each point in graph represent data obtained from a single worm. Bars are means ± s.d. Arrows on top indicate statistical significance with *p<0*.*05* when compared to N2 control.
(TIF)

S3 Fig Effects of silencing the expression of BCL-XL variants in various transgenic strains and control worms. Typical images of DAPI-stained cells with mitotic region and transition zone length in C. elegans gonads.
(TIF)

S4 Fig Effects of silencing the expression of BCL-XL variants in various transgenic strains and control worms. Typical images of CED-1:GFP and DAPI-stained cells.
(TIF)

S1 Table Vector design and transgenic strains. (PDF)
