# Supplementary figures and images for: Expression of human Bcl-xL (Ser49) and (Ser62) mutants in Caenorhabditis elegans causes germline defects and aneuploidy
Source: PLoS One. 2017 May 8;12(5):e0177413. doi: 10.1371/journal.pone.0177413 (PMC5421811; doi:10.1371/journal.pone.0177413)

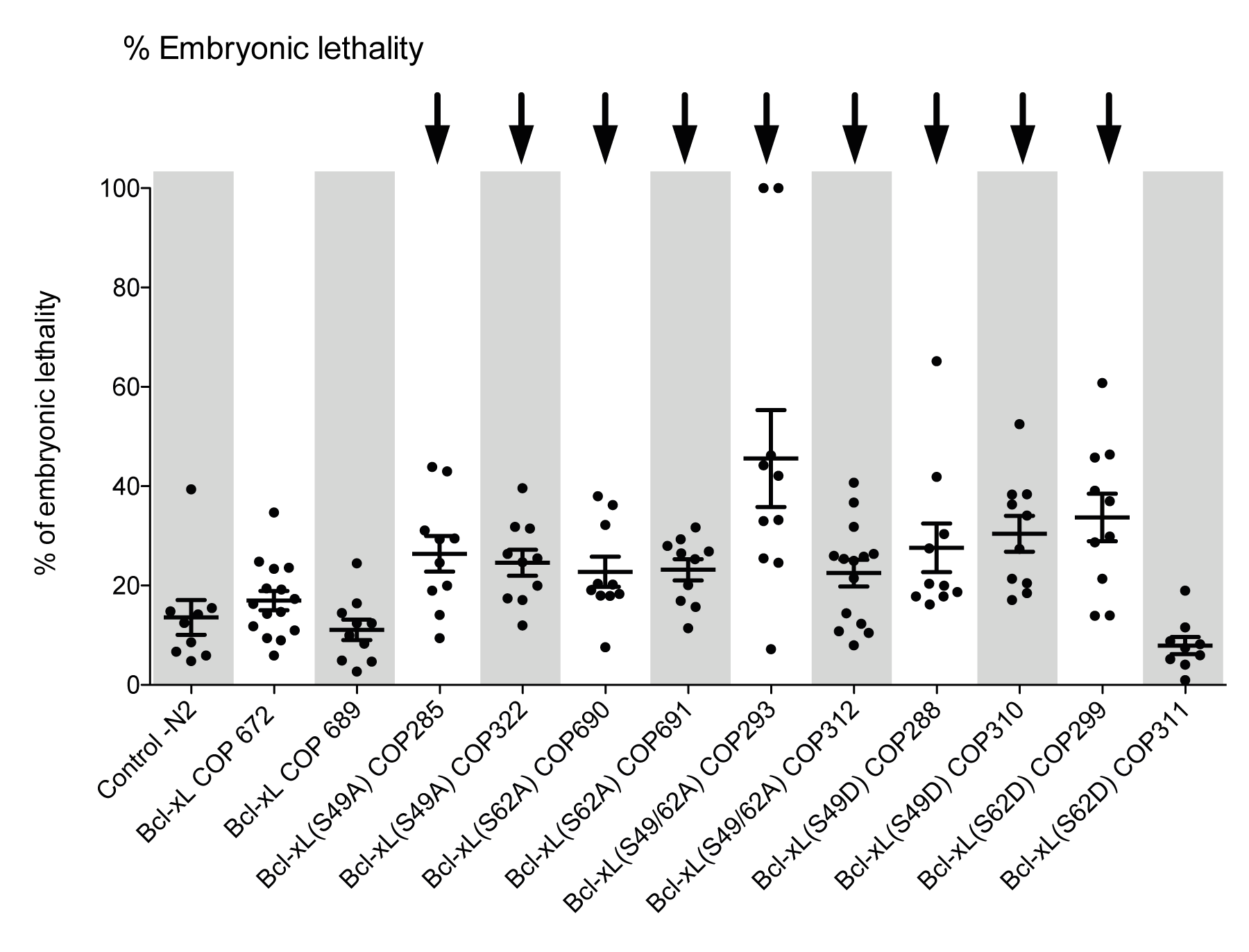

Supplement: S1 Fig — Percentages of embryonic lethality in various transgenic strains and control worms. Each point show in the graphs represents data obtained from a single worm. Bars are means ± s.d. Arrows on top indicate statistical significance with p<0.05 when compared to N2 control. (TIF) [file pone.0177413.s001.tif]

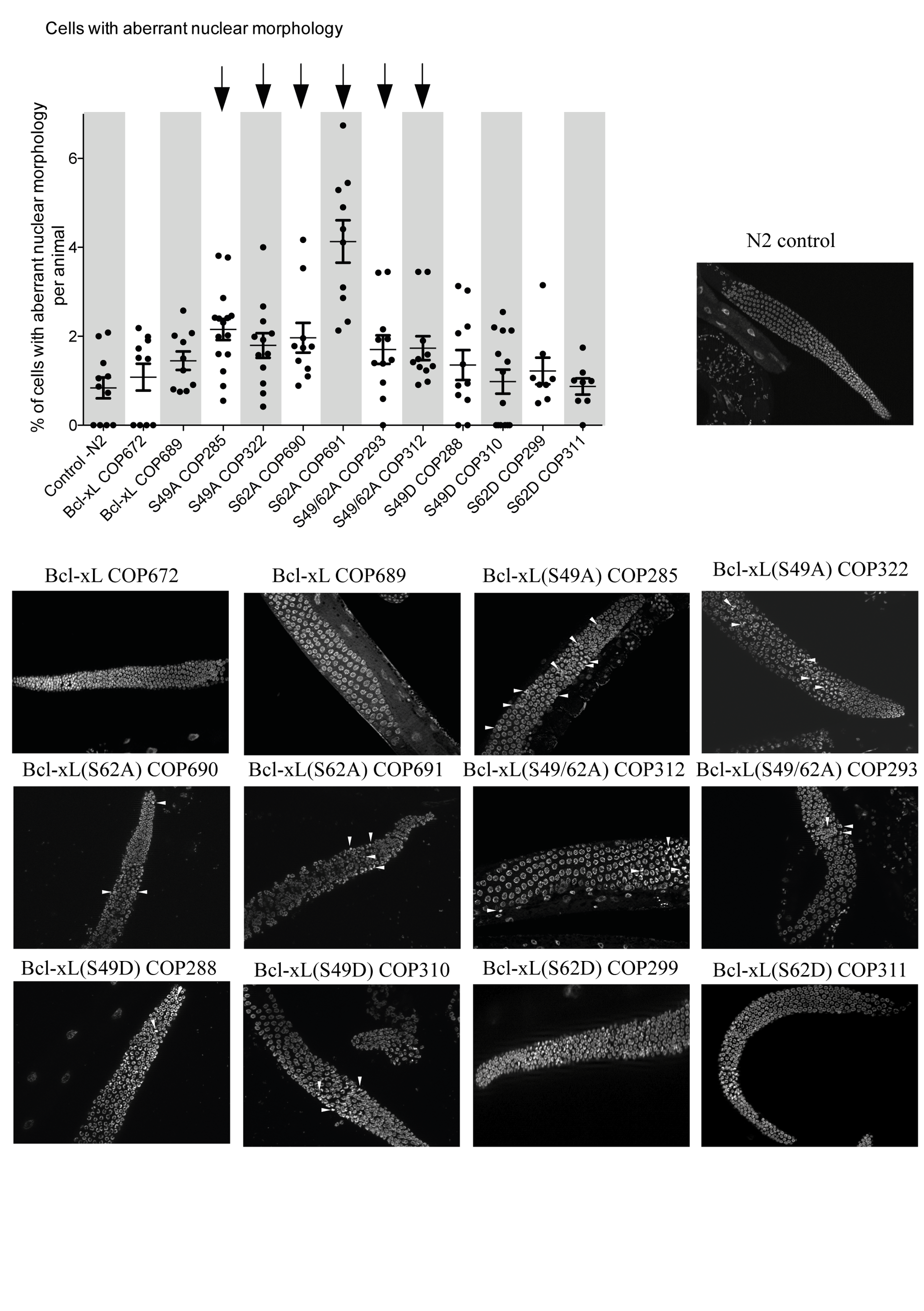

Supplement: S2 Fig — Graph showing the percentage of aberrant cells per worms and images of DAPI-stained germlines of various trangenic strains and control worms. Each point in graph represent data obtained from a single worm. Bars are means ± s.d. Arrows on top indicate statistical significance with p<0.05 when compared to N2 control. (TIF) [file pone.0177413.s002.tif]

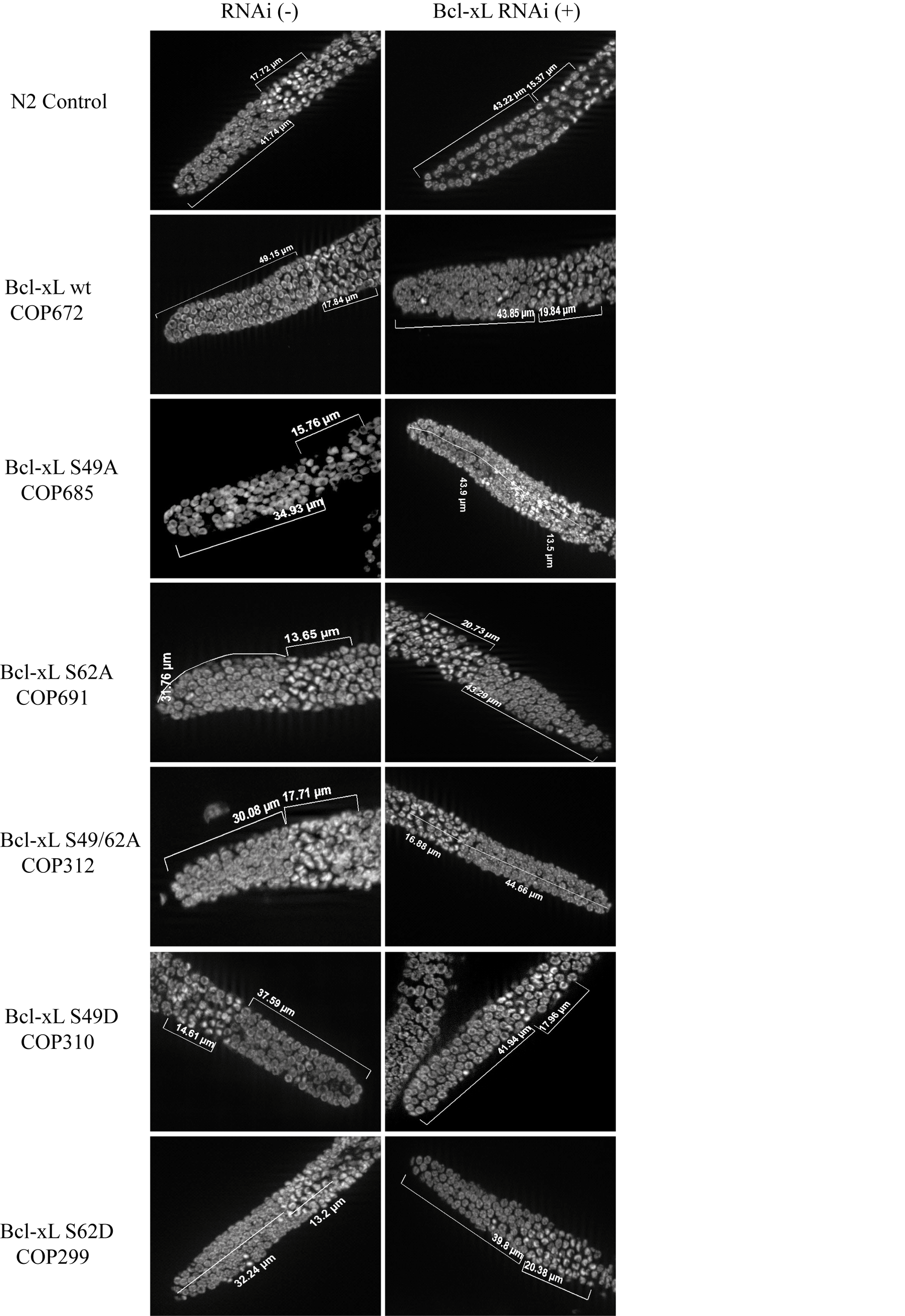

Supplement: S3 Fig — Typical images of DAPI-stained cells with mitotic region and transition zone length in C. elegans gonads. (TIF) [file pone.0177413.s003.tif]

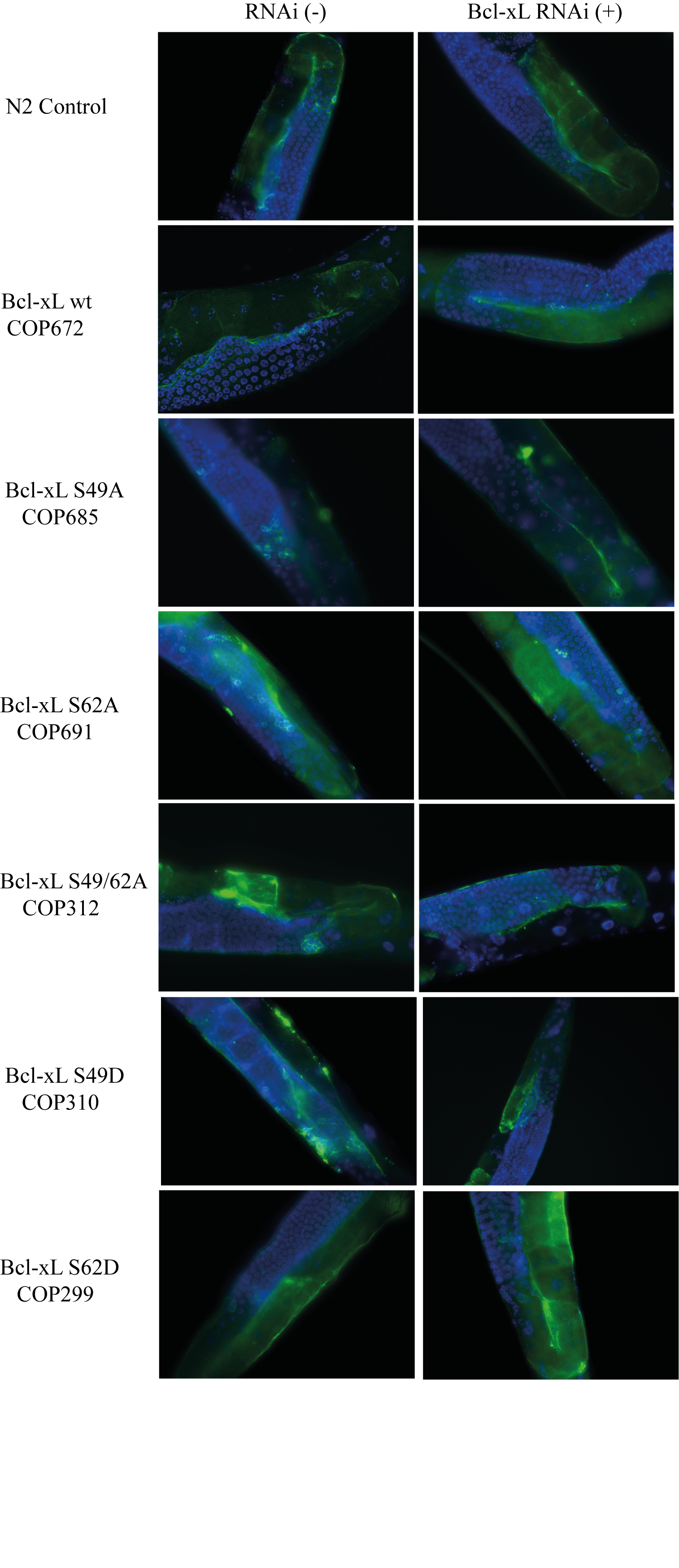

Supplement: S4 Fig — Typical images of CED-1:GFP and DAPI-stained cells. (TIF) [file pone.0177413.s004.tif]
