# Supplementary material for: Expression of human Bcl-xL (Ser49) and (Ser62) mutants in Caenorhabditis elegans causes germline defects and aneuploidy
Source: PLoS One. 2017 May 8;12(5):e0177413. doi: 10.1371/journal.pone.0177413 (PMC5421811; doi:10.1371/journal.pone.0177413)
Supplement: S1 Table — (PDF) [file pone.0177413.s005.pdf]

S1 Table Vector design and transgenic strains

Vector design	Transgenic strain ID
unc-119 ced-9 promoter / HA-Bcl-xL wt / 3'UTR	COP287, COP297,COP672,COP689
unc-119 ced-9 promoter / HA-Bcl-xL(S49A) / 3'UTR	COP285, COP286,COP322
unc-119 ced-9 promoter / HA-Bcl-xL(S62A) / 3'UTR	COP355, COP690,COP691
unc-119 ced-9 promoter / HA-Bcl-xL(S49D) / 3'UTR	COP288, COP298,COP310
unc-119 ced-9 promoter / HA-Bcl-xL(S62D) / 3'UTR	COP289,COP290,COP295, COP299,COP311
unc-119 ced-9 promoter / HA-Bcl-xL(S49/62A) / 3'UTR	COP291,COP293,COP300, COP301,COP312
unc-119 ced-9 promoter / HA-Bcl-xL(S49/62D) / 3'UTR	none
